# Atmospheric impacts of the strongest known solar particle storm of 775 AD

**DOI:** 10.1038/srep45257

**Published:** 2017-03-28

**Authors:** Timofei Sukhodolov, Ilya Usoskin, Eugene Rozanov, Eleanna Asvestari, William T. Ball, Mark A. J. Curran, Hubertus Fischer, Gennady Kovaltsov, Fusa Miyake, Thomas Peter, Christopher Plummer, Werner Schmutz, Mirko Severi, Rita Traversi

**Affiliations:** 1 Physikalisch-Meteorologisches Observatorium Davos World Radiation Center, Davos, Switzerland; 2 Institute for Atmospheric and Climate Science, Swiss Federal Institute of Technology Zurich, Zurich, Switzerland; 3 Space Climate Research group, University of Oulu, Finland; 4 Sodankylä Geophysical Observatory, University of Oulu, Finland; 5 Department of the Environment, Australian Antarctic Division, Kingston, Australia; 6 Antarctic Climate and Ecosystem Cooperative Research Centre, University of Tasmania, Hobart, Australia; 7 Climate and Environmental Physics, Physics Institute and Oeschger Centre for Climate Change Research, University of Bern, Bern, Switzerland; 8 Ioffe Physical-Technical Institute RAS, St. Petersburg, Russia; 9 Institute for Space-Earth Environmental Research, Nagoya University, Nagoya, Japan; 10 Institute for Marine and Antarctic Studies, University of Tasmania, Hobart, Australia; 11 Dept. of Chemistry “Ugo Schiff”, University of Florence, Florence, Italy

## Abstract

Sporadic solar energetic particle (SEP) events affect the Earth’s atmosphere and environment, in particular leading to depletion of the protective ozone layer in the Earth’s atmosphere, and pose potential technological and even life hazards. The greatest SEP storm known for the last 11 millennia (the Holocene) occurred in 774–775 AD, serving as a likely worst-case scenario being 40–50 times stronger than any directly observed one. Here we present a systematic analysis of the impact such an extreme event can have on the Earth’s atmosphere. Using state-of-the-art cosmic ray cascade and chemistry-climate models, we successfully reproduce the observed variability of cosmogenic isotope ^10^Be, around 775 AD, in four ice cores from Greenland and Antarctica, thereby validating the models in the assessment of this event. We add to prior conclusions that any nitrate deposition signal from SEP events remains too weak to be detected in ice cores by showing that, even for such an extreme solar storm and sub-annual data resolution, the nitrate deposition signal is indistinguishable from the seasonal cycle. We show that such a severe event is able to perturb the polar stratosphere for at least one year, leading to regional changes in the surface temperature during northern hemisphere winters.

Solar energetic particle (SEP) events have been shown to affect the Earth’s atmosphere due to the depletion of stratospheric ozone^1^,^2^ and therefore to be potentially hazardous for life on Earth due to the following increase of solar ultra-violet (UV) irradiance at the surface^3^. Model simulations suggest that such severe events may also potentially affect the surface weather^2^. An exceptionally strong^4^ cosmic-ray event of 774–775 AD was discovered recently^5^ in radiocarbon ^14^C measured in annually dated tree rings^6^,^7^,^8^ and confirmed by ^10^Be and ^36^Cl cosmogenic isotopes measurements^9^,^10^,^11^. Although various scenarios were initially proposed, it is concluded now^6^,^9^ that the event was caused by SEPs. The event was short in duration (possibly including several pulses) with a very hard energy spectrum, as estimated using the ratio of different cosmogenic isotopes^9^. It was 40–50 times stronger than the largest directly observed event (23-Feb-1956)^6^,^9^, making the event the strongest known during the last 11 millennia^4^,^12^. It is so distinct in the ^10^Be data (Fig. 1 and Fig. S1 in Supplementary Materials (SM)) that it serves as a tie point for ice core dating^10^. Accordingly, this event forms a benchmark of the worst-case scenario in the SEP influence on the Earth’s environment. Here we model this event and its atmospheric effects and compare it to extensive ice core data.

## Results and discussion

### Impact on ice core ^10^Be

**Figure 1:**
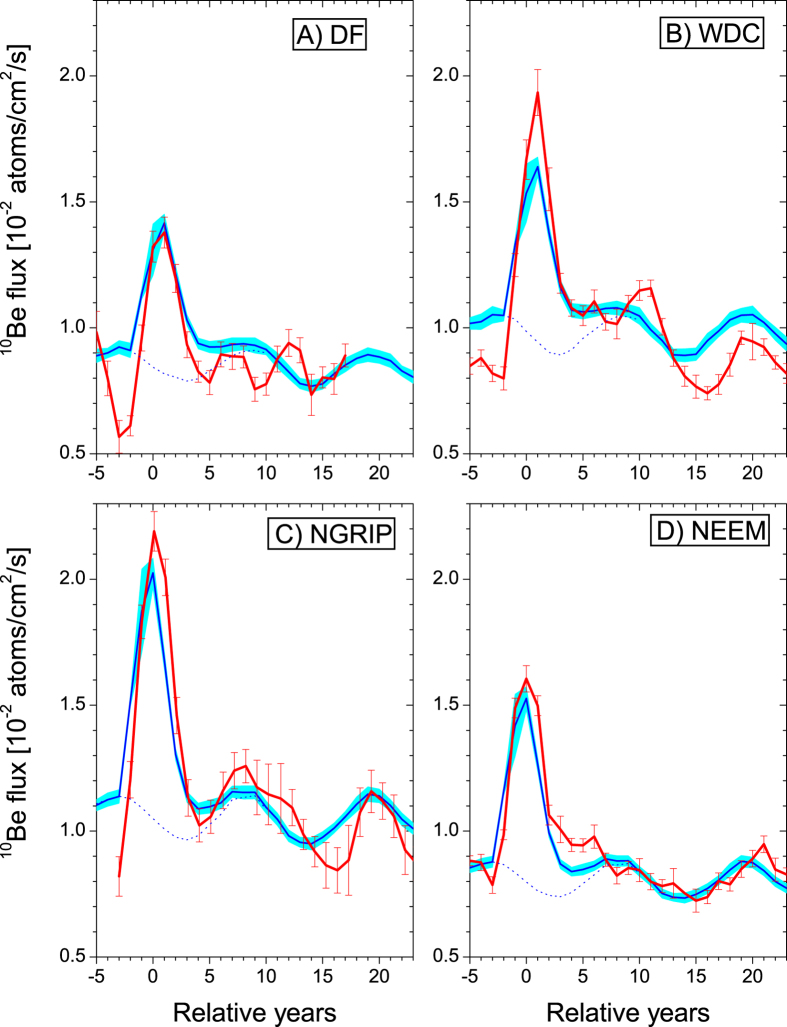
Annual depositional fluxes of ^10^Be at four sites analyzed for the period around 775 AD. Panels A through D correspond to Dome Fuji (Antarctica), WDC (Antarctica), NGRIP (Greenland) and NEEM (Greenland), respectively. Red lines with error bars depict the measured data, while blue lines depict the modeled ^10^Be flux for the boreal Autumn scenario. Blue shaded area represents the 1σ model uncertainty. All curves are 3-yr running means and scaled to match the GCR-induced level for years 7–17. Years are given relative to the peak year. See Supplementary Tables S1–2 online for the sites and scenarios details.

We used published quasi-annual ^10^Be measurements (see Fig. S1 and Table S1 in SM) from Greenland (NGRIP and NEEM) and Antarctic (DF and WDC/WAIS) ice cores for model validation. Because of dating uncertainties^10^ we adjusted all observations to match the peak at relative year 0. For all sites, we computed the expected ^10^Be deposition for four injection scenarios and 5 ensemble members (see Methods section). Due to model uncertainties^6^,^13^, we rescaled simulated ^10^Be fluxes by an ad-hoc factor of 0.79–1.88 (Table S2 in SM) to match the ^10^Be data for relative years 7–17 when the event related peak ceased and the production was determined by galactic cosmic rays (GCR). The model underestimates deposition in Antarctica (scaling factors 1.42 and 1.88 for DF and WDC/WAIS, respectively), but yields a better agreement in Greenland (1.00 and 0.79 for NGRIP and NEEM). This is likely related to a simplified deposition scheme that isn’t able to resolve regional distribution in deposition flux sufficiently. However, the model results are representative for a hemispheric estimate, which after local rescaling is sufficient for the analysis of relative annual variability, as used in our study. Figure 1 shows that the scaled modelling results reproduce well the 11-year solar cycle, both in phase and amplitude, except for DF, where high depositional noise in this very low accumulation rate area due to wind reworking may obliterate the 11-year cycle. The model also well reproduces the scaled SEP-induced peak, i.e., the integrated ^10^Be SEP-signal is consistent with the data within 10–20% (Table S2).

Atmospheric transport of ^10^Be produced in the stratosphere depends on the large-scale stratospheric circulation, which in turn depends on the season when the production occurred. Based on ^14^C data analysis^7^, there were some estimates of the potential season of occurrence for this event, favoring Spring. Here we also addressed this question on a basis of ^10^Be analysis by simulating the event to appear in different seasons (see Methods and Fig. S2). Analyzing the Pearson correlation between the measured and modeled signals and the shape of the peak, we found that the event occurred most likely in boreal Autumn, whereas Spring is less likely according to the Greenland data (Supplementary Table S2). Note, that our estimate is strongly related to the shape of the peak in ^10^Be observations in both hemispheres. If the seasonal timing of the event is also affected by non-equidistant sampling in time in the ice cores^9^, our conclusions on the event timing may be incorrect. Further results presented in this paper are mainly based on our Autumn estimate, but for comparison of the possible climate/chemistry effects we also performed simulations for the Spring estimate.

### Impact on ice core nitrate

A strong SEP event can affect atmospheric chemistry through the production of HO_x_ (H + OH + HO_2_) and NO_x_ (NO + NO_2_) from N_2_ and O_2_ ionization, mainly in the polar stratosphere and mesosphere^1^,^2^,^14^. Since NO_x_ is a precursor for atmospheric nitrate, it was suggested that strong SEP events can be detected as sharp spikes in records of nitrate in polar ice^15^. However, this was debated both experimentally for the Carrington event^16^ and theoretically^17^. If a nitrate record was indeed a proxy for strong SEP events of the past^11^,^18^, the extreme event of 774 AD would be clearly identified in polar nitrate series. Accordingly, an analysis of an extreme SEP event is a crucial point to ultimately resolve this question.

**Figure 2:**
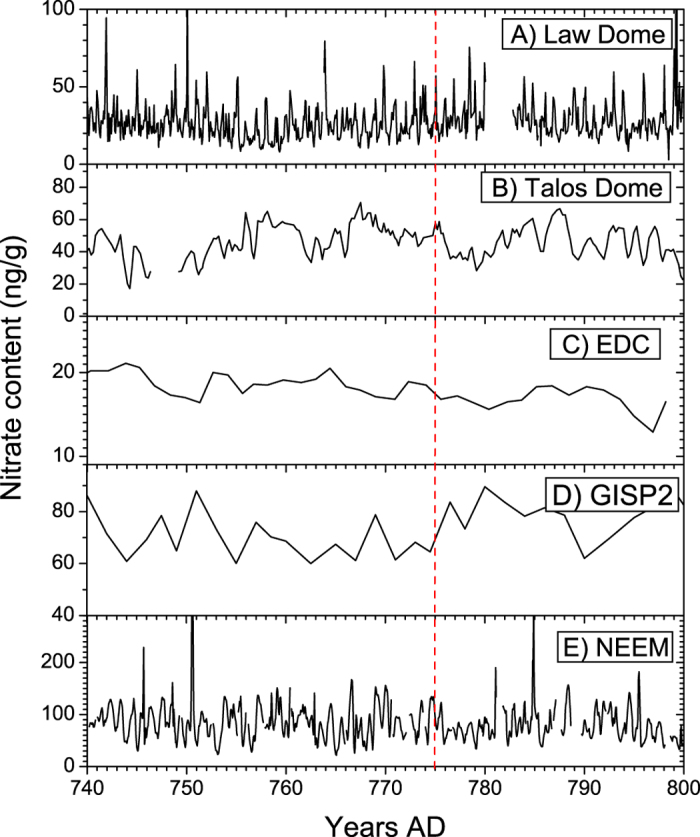
Measured concentrations of nitrate in several polar ice cores around 775 AD (see Table S1). Panel A: Law Dome, data with about monthly resolution; dating uncertainty is ±7 year. Panel B: Talos Dome, data with quarterly resolution. Panel C: Annual data from EPICA Dome C. Panel D: data from GISP2. Dating uncertainty of these three data sets can be also on the order of a several years. Panel E: NEEM, data with about 20 samples per year time resolution; note that the NO_3_ data is from the NEEM main core, while the ^10^Be peak at 774–775 was derived on a shallow core close-by (NEEM-S1). The relative dating uncertainty of these two neighboring cores should not be more than a few years and clearly smaller than 7 years. Within this uncertainty no NO_3_ peak is found in the NEEM main core. The vertical red dashed line depicts the proposed year of the SEP event.

**Figure 3:**
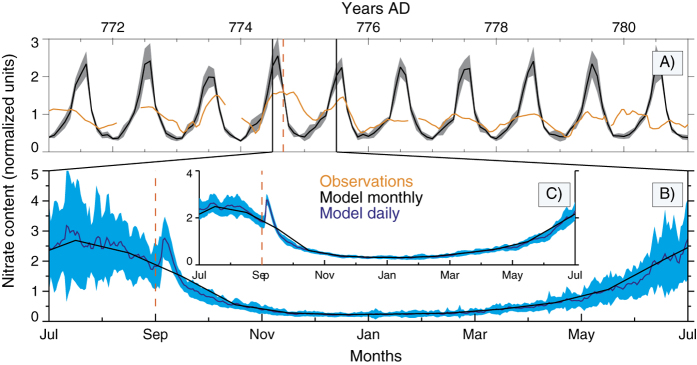
Measured and modelled deposited nitrate in the polar region, divided by the mean of the time-series. Panel A: 10 years of observational and monthly mean modelled data for the Greenland NEEM location. Panel B: zoom of panel A for the period July 774 – July 775 AD representing modelled monthly and daily mean data. Panel C: same as panel B but averaged over the whole polar cap (70–90°N). Grey and blue shaded areas represent modelled monthly mean and daily mean 1σ uncertainty, respectively. The event is assumed to occur on 01-Sep-774 (as denoted by the vertical red dashed line).

We investigate this possibility with both observational and modelled nitrate content in polar ice cores at annual and sub-annual resolutions (see Methods). No clear spikes potentially related to SEP-events were found in any of the 5 series analyzed around 774 AD even considering the possible dating uncertainties of few years (Fig. 2). There are small peaks in the Law Dome and Talos Dome series, but they are indistinguishable from the variability in the records. Also it is worth to mention that no obvious spike is found in 5-cm resolution NGRIP data around 775 AD (not shown, A. Svensson, private communication, Aug. 2016). While the time resolution of GISP, EPICA Dome C and Talos Dome may be too coarse to distinguish such a spike^19^, the Law Dome and NEEM series, with 12–20 samples per year, could have sufficient resolution to resolve a hypothetical spike. At the NEEM site both ^10^Be and NO_3_^−^ data are available, although derived from two separate neighboring cores with relative age uncertainties of the order of a few years. Thus for further illustration we use an analysis of the NEEM data (Fig. 3). Figure 3A shows the variability of the observed and modelled monthly deposition of nitrate for the NEEM location. The modelling results show a pronounced seasonal cycle caused by NO_x_ emissions from other natural sources. The seasonal amplitude is in reasonable agreement with the observations given prevalent reworking of the snow pack after deposition, whereas the observed phase somewhat diverges, potentially due to intra-annual uncertainties of the sample dating due to a seasonal cycle in snow accumulation. In Fig. 3A, there is no clear signal in the nitrate concentration, neither in simulated nor measured series, that could be associated with the extreme SEP event. Figure 3B shows a one-year subsample of the modelled daily data for the same location. It shows a noticeable increase of deposited nitrate in September 774 AD, when the simulated event was placed, related to the tropospheric/lower stratospheric production. However, it is hardly distinguishable from the seasonal variability considering its uncertainty, defined by local meteorology, and cannot be discerned anymore in modeled monthly averages. This conclusion is valid also for other analyzed locations (not shown). A larger-scale picture is provided in Fig. 3C, depicting the same data as in panel B but averaged over the entire northern polar cap. A more pronounced nitrate increase can be seen, but it is still too small and short to be clearly distinguishable in observed and modelled monthly averaged time-series.

Thus, based on modeling results and measurements in different ice cores, we conclude that, even with a fine temporal resolution and excluding post-depositional processes, a nitrate signal of the SEP event of 774–775 AD is not observable in polar ice core records. Because of the relatively soft spectrum (even for such an extreme event), SEPs produce NO_x_ mostly in the polar stratosphere, from where it takes, depending on meteorological conditions^20^, several months to reach the troposphere. SEP-induced nitrate production in the troposphere would have a direct effect on ice core concentrations, but its fraction is very small (only several percent). Thus, no immediate big spikes can be expected in the nitrate records even in association with such an extreme SEP event. A longer spike lasting several years, as for ^10^Be, is also unlikely for nitrate, since in contrast to ^10^Be, whose production is defined by cosmic rays, nitrate has other natural sources with a pronounced seasonal variability. If such an extreme SEP event cannot produce a distinguishable peak in polar nitrate, it is very unlikely that smaller events would do^16^. This, however, does not necessarily exclude a possibility to use nitrate records as proxy for centennial GCR variability^21^. In general, our model shows a statistically significant increase (up to 50%) in deposited nitrate during the first month after the event in high-latitude regions (above 45°), obtained as monthly mean differences between runs with and without the event (See Methods). The effect, which is more pronounced for the southern hemisphere, (Fig. S3 in SM) can extend for several months due to a gradual transfer of the enhanced nitrate from the stratosphere.

### Impact on atmospheric chemistry, temperature, and dynamics

**Figure 4:**
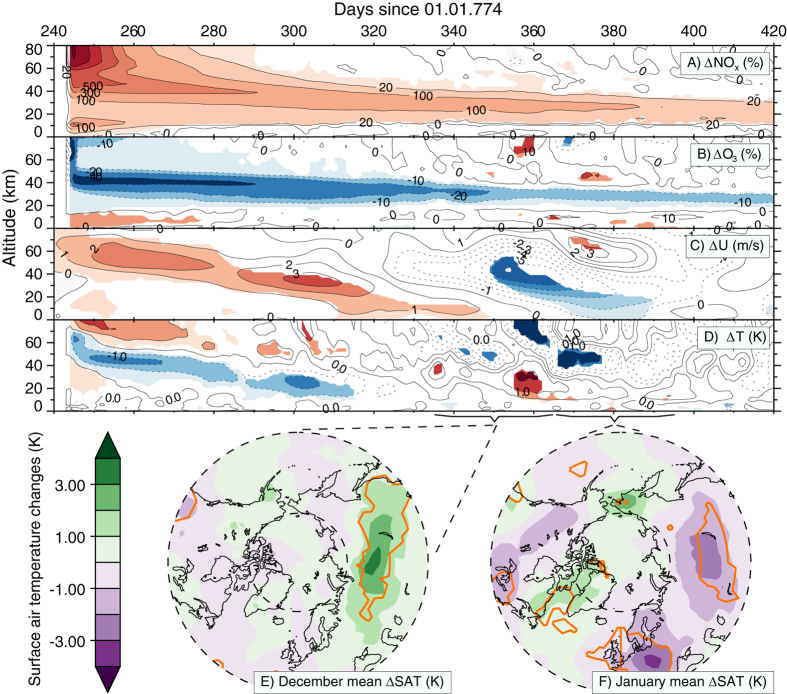
Atmospheric effects due to the SEP event on 01-Sep-774 (day 244). Panels A through D: NO_x_, O_3_, zonal wind (U) and temperature (T) anomalies, respectively, averaged over the northern polar region (70–90°N for NO_x_, O_3_ and T and 50–70°N for U) and averaged over all 30 ensemble members. Zonal wind changes are shown as 20-day running means. Colored areas are significant at a 95% confidence level. Panels E, F: Monthly mean surface air temperature (SAT) changes (K) in December 774 AD and January 775 AD due to the event. The orange contours indicate significance at the 95% confidence level. Dashed lines mark 40°N and 70°N latitudes. (Maps are plotted using IDL version 8.2, http://www.harrisgeospatial.com/ProductsandSolutions/GeospatialProducts/IDL.aspx).

The potential for SEP events to influence the Earth’s atmosphere has been discussed for a long time based on analysis of satellite observations and numerical modelling^1^,^2^. Previous modelling studies, however, analyzed smaller or hypothetical events^2^,^3^. Typically, the initial HO_x_ and NO_x_ formation in the stratosphere leads to additional ozone depletion in NO_x_ and HO_x_ driven catalytic cycles followed by a decrease in temperature. This temperature anomaly then modulates the Polar-Night Jet Oscillation (PJO) (similarly to the solar 11-year UV variation effects^22^) and creates a positive and, later, a negative wind anomaly both propagating down. The overall impact is stronger in the winter hemisphere because of air isolation by the polar vortex and the fact that coupling between polar stratosphere and troposphere is the strongest during winter. Figure 4A–D illustrates all these effects for the 775 event as a difference in northern polar NO_x_, ozone, temperature, and zonal wind between the modelling runs with and without the event in ionization rates (see Methods). Further propagation of the anomaly down to the troposphere is described by the so-called “top-down mechanism”, which results in the modulation of the tropospheric circulation with local weather consequences^23^. In accordance with this, the model suggests that monthly mean near-ground land temperatures differ by up to 4 K (Fig. 4E–F) between the ensemble runs with and without the event. For December 774 AD the model yields an acceleration of the tropospheric mean flow in the northern hemisphere and thus a more pronounced meridional circulation causing warming over Siberia. For January 774 AD, conversely, the model predicts more significant (orange contour) cooling in Siberia, Europe and Canada caused by the negative stratospheric zonal mean zonal wind anomaly. The reduction of the zonal mean total ozone persists for at least one year (see Supplementary Fig. S4). The maximum decrease in the globally averaged total ozone is reached during the second month after the event at a level of −8.5% for the ensemble mean. Thomas *et al*.^3^ calculated potential effects of SEP events on life on Earth. From their estimates we can conclude that a −8.5% decrease in total ozone, as found here for the 774–775 event, would likely have only a moderate biological impact.

The Autumn scenario analyzed here provides the most favorable conditions to perturb the northern stratospheric vortex with the following downward propagation, as there is still sunlight to enhance a stratospheric temperature anomaly. For completeness we also performed an assessment of the climate effect of an event of the same size but occurring in spring 775, as suggested by Güttler *et al*.^7^. Analysis of this scenario showed a similar global total ozone decrease and that by the northern winter next year there is still a statistically significant −10% ozone anomaly in the polar stratosphere. Polar stratospheric ozone anomaly can be considered as a forcing for the subsequent dynamical effects, and the Spring scenario therefore provides a several times weaker forcing compared to the Autumn scenario (~−20–40% during vortex formation, Fig. 4B). Nevertheless, our results suggest that this is still enough to modulate the PJO and, hence, the surface weather. Similar ~−10% forcing and dynamical effects were obtained by Calisto *et al*.^2^ for the weaker (~2.5 times) hypothetical event. Therefore, we can conclude that regardless of whether the SEP event of the 775 AD-like size is placed in Spring or Autumn, there are still significant (albeit not catastrophic) changes to chemistry and dynamics of the northern hemisphere. Note, however, that the downward propagation of the signal is always subject to the current state of the vortex given by the stratospheric wave-mean flow interactions of a particular year. Comparison with the results of Calisto *et al*.^2^, who used a similar model, Autumn season, but present day conditions, also allows us to conclude that the stratospheric SEP effects on chemistry are not dramatically affected by the changing climate, since we also show ~2.5 times larger response in ozone and NO_x_ for the Autumn scenario.

## Conclusions

Based on our 3-D modelling results and observational data analysis, we conclude that the SEP event of 774–775 AD was able to decrease the stratospheric ozone for more than one year and thus to modulate the surface weather. In contrast to previous hypothetical estimates^3^, our analysis is based on the latest event strength assessment^6^,^9^, which, as we show, is also supported by good agreement between modelled and observed ^10^Be deposition variability. Similarly to prior conclusions^16^,^17^, our results suggest that even an extreme SEP event cannot be resolved in ice core nitrate time-series devaluating it as a potential proxy for SEP events. Since the analyzed SEP event is the strongest known in the Holocene^4^,^12^, our results can serve as a realistic upper bound (worst-case scenario) of the possible effect of SEP events on the atmosphere.

## Methods

### Chemistry-climate model

We used the chemistry-climate model (CCM) SOCOL v3.0, which mainly consists of the dynamical core MA-ECHAM5 and the chemical core MEZON, interacting with each other every 2 modelling hours. The model has 39 vertical levels between Earth’s surface and 0.01 hPa (~80 km). For the present study, we used the horizontal resolution of about 2.8° × 2.8° (T42). The detailed description of the model is given by Stenke *et al*.^24^. Compared to the model version described by Stenke *et al*.^24^, the present model underwent some further improvements such as the more detailed tropospheric isoprene chemistry^25^, interactive lightning NO_x_ parameterization^25^, parameterizations of energetic particles effects of different origin including GCR and SEP^2^ and the extra-heating parameterization allowing a precise simulation of the 11-year cycle in the heating rates^26^. The main boundary conditions driving the model are the prescribed fields of sea surface temperature (SST), sea ice coverage (SIC), stratospheric and tropospheric aerosols, spectral solar irradiance (SSI) and emissions of greenhouse gases (GHG) and ozone-destroying substances (ODS). It should be noted that continental surface temperatures respond to the SEP-induced forcing (Fig. 4E–F). The fact that SST and SIC are prescribed does not compromise the model results, because ocean effects would be essentially the same with interactive or prescribed ocean under such a short-lived forcing (given the large heat capacity of the ocean mixed layer).

In the stratosphere, ^10^Be gets readily attached to stratospheric aerosols, but in the model is transported like a gas without additional gravitational settling. This is partly justified by the small size of the background aerosol particles with average sedimentation speeds less than 250 m per month in the stratosphere. Removal of ^10^Be from the stratosphere is organized using predefined effective dry deposition velocities in the lowest model layer for different types of surfaces (0.6, 0.8, 0.1 cm/s for land, sea, and ice, respectively). Although this represents a very simplified approach, as aerosol attached ^10^Be is affected strongly by wet deposition, model estimates agree with the relative global tropospheric burden (~12%) from the detailed modelling by Heikkilä *et al*.^27^ (11–13%). The model also considers dry deposition of several chemical gaseous species including species from the nitrogen group (NO, NO_2_, HNO_3_, N_2_O_5_ and PAN) using predefined dry deposition velocities^24^. In addition, the removal of the soluble HNO_3_ by tropospheric precipitation is represented by a constant removal rate of 4 × 10^−6^ s^−1^. Here, we analyze the sum of the modelled wet and dry deposition fluxes of HNO_3_.

### ^10^Be, NO_x_ and HO_x_ production rates

We assumed that the event of 774–775 AD had a very hard energy spectrum similar to that of the strongest directly recorded SEP event of 23-Feb-1956, which caused the strongest tropospheric ionization over the last 60 years^28^. The assumption of the hard spectrum is validated by a recent analysis^29^ of ground level enhancement (GLE) events. The spectrum was taken in the form of the Band-function as parameterized by Tylka and Dietrich^30^, but scaled up by a factor of 45 as obtained from analyses^6^,^9^ of cosmogenic isotope data of ^14^C, ^10^Be and ^36^Cl. The total fluence of SEPs with energy above 30 MeV for this event is estimated as *F*_*30*_ = 4.5 × 10^10^ cm^−2^. The *F*_*200*_ fluence (>200 MeV)^31^ was about 8 × 10^9^ cm^−2^. An instantaneous (1-day duration) injection of SEP into the atmosphere with isotropic flux was assumed.

For the ^10^Be production we used the CRAC:10Be model^32^ which simulates the cosmic-ray induced atmospheric cascade using a direct Monte-Carlo method. The model yields the 3D (geographical coordinates and altitude) time-dependent rates of ^10^Be production in the atmosphere. Further transport of beryllium was treated by the CCM SOCOL. The simulated ^10^Be background concentration (see Fig. S5 in SM) appeared to be in good agreement with the recent results^33^ obtained using the same production rates by GCR, with slightly higher stratospheric concentrations in the polar regions and lower concentration in the tropics. Such redistribution, among other modelling differences, can be attributed to the fact that SOCOL generally shows faster Brewer-Dobson circulation than other models. On the other hand, Delaygue *et al*.^33^ also noted that their model underestimates maximum concentrations compared to observations.

For cosmogenic production of nitrate, we first calculated the atmospheric ionization rate by energetic particles using the CRAC:CRII model^34^. Then the ionization rate was used as an input for the CCM SOCOL which treats the chemical and transport changes. Other sources of nitrate in the model are lightning, biomass burning and stratospheric decomposition of N_2_O. Note that we do not consider any post-depositional chemistry and re-emission from the snowpack.

### Design of numerical experiments

For our numerical experiments, we aimed at reproducing the conditions of the 8^th^ century as close as possible despite very limited measurement data being available. For SST, which is the main climate driver for our model, we used a reconstruction derived from a set of global proxy data^35^. As the mid-20^th^ century is estimated to have approximately the same temperatures in this reconstruction as in the end of the 8^th^ century, we set all other boundary conditions constant at the 1960-year level, keeping the interannual variability and disabling all anthropogenic sources of aerosols, GHG and ODS. We assumed only background stratospheric aerosol concentrations, since there were no large volcanic eruptions at that time^10^. The SEP event occurred on a background of permanent flux of galactic cosmic rays (GCR) which is subject to solar modulation with roughly 11-year periodicity. The level of cosmic ray modulation was moderate (ϕ ≈ 500–550 MV) during that period^36^,^37^, similar to that during the 1970’s. Accordingly, in order to model the effects of the GCR background and SSI we assumed a moderate 11-year cycle with the modulation parameter ϕ varying between 400 MV (solar minimum) and 700 MV (solar maximum) with the phases derived from the ^10^Be observations (Fig. 1). Both GCR and SEP are additionally shielded by the geomagnetic field in the vicinity of Earth. We modelled this effect as well using the geomagnetic field for the epoch 750–800 AD from a recent archeomagnetic reconstruction^38^ and calculating the effective geomagnetic rigidity cutoffs for each location on Earth applying an eccentric dipole approximation.

After a 10-year spin-up we initiated a 5-member 30-year long ensemble run from 771 AD to 800 AD. Four ^10^Be injection scenarios were modelled with identical parameters except for the date of particle injection, being 01-June, 01-September, 01-December of the year 774 AD and 01-March of the year 775, respectively. For the nitrate deposition and chemistry analysis we performed a 30-member ensemble run for the years 774–775 with the ionization event set on the 01-September of 774 AD and a control run with the same 30 members but without the event. Then, in order to isolate the atmospheric effects caused by the event, we subtracted control runs from runs with the event and calculated the statistical significance of the difference. In addition, we also performed the same procedure but for the event initiated on the 01-March of 775 AD and 20 ensemble members.

### ^10^Be and Nitrate observations

Here we use data of the depositional ^10^Be flux with quasi-annual resolution from four different ice cores (Fig. 1 and Table S1 in supplements): two from Antarctica (Dome Fuji and WDC/WAIS) and two from Greenland (NGRIP and NEEM-S1). The depositional flux is obtained as a product of the ^10^Be concentrations measured in ice cores using the Acceleration Mass Spectrometry (AMS) method (see details in Beer *et al*.^13^) and snow accumulation rates which are yielded by flow model (Dome Fuji) or annual layer counting and thinning function model results (WDC, NEEM, NGRIP). The absolute dating uncertainties at 775 AD are within ±7 years, but since the ^10^Be peak is clearly seen in all the data series, we have adjusted timing of the data to match the peaks^10^ (See Table S2 in SM), so that the year 775 AD is set as year 0 in our results.

The nitrate records used in this work were derived from four ice cores (Supplementary Table S1): three from Antarctica (Law Dome, Talos Dome, EPICA Dome C) and two from Greenland (NEEM main core, GISP2). Nitrate measurements were carried out by different analytical methods for different ice cores. A classical Ion Chromatography method on discrete samples was applied to the Law Dome and GISP2 ice cores^39^ whereas for the other three cores, a continuous (NEEM main core) or a quasi-continuous method (Talos Dome, EDC) were used, coupling the measuring devices to a melting system. In particular, a spectrophotometric Continuous Flow Analysis (CFA) method was employed for the NEEM main core^40^ and two Fast Ion Chromatography (FIC) methods for the EDC and Talos Dome cores^41^. The high annual accumulation rate at the Law Dome (and NEEM) sites allows annual layer counting; NEEM dating has been recently revised further according to Sigl *et al*.^10^. Talos Dome and EDC cores were dated through an accurate match of volcanic signatures with the WDC/WAIS core, according to Sigl *et al*.^10^ providing a similar dating uncertainty (±2 years).

### Uniqueness of the event in ^14^C data

Presently, only two extreme SEP events have been discovered in the past: around 775 AD^5^ and 994 AD^42^. It is unlikely that an event stronger than that of 775 AD will be found for the Holocene. The Japanese team^5^ performed a systematic search for the events, starting from annually resolved Δ^14^C measurements around the sharpest peak increase over the Holocene in the INTCAL dataset (~0.4‰/year) around 775 AD and found the event^5^ (note that the 775 AD peak is clearly distinguishable in the decadal Δ^14^C INTCAL dataset - see Usoskin *et al*.^4^). Then they analyzed the second peak (~0.2‰/year) and discovered a smaller event at 994 AD^42^ which is barely seen in the INTCAL data. After that, the team has performed high-resolution Δ^14^C measurements around four other peaks (~0.3‰/year) over the last several millennia and found no additional events^12^. Overall, there are 15 intervals with increase rates ≥0.3‰/year in the INTCAL13 dataset over the Holocene^14^, out of which five have been measured with high resolution leading to the discovery of the 775 AD event, while four others were not related to SEP events. Only two intervals over the Holocene contain increase rates comparable to that of 775 AD (~0.4‰/year). Even if these intervals correspond to the SEP-like sharp events, they should not be much stronger than the event of 775 AD. In particular, an event twice as strong as that of 775 AD would have produced a large spike in the INTCAL data which could not be missed^4^. Thus, we conclude that even though the event of 775 AD is not necessarily unique, it can reliably serve as the worst case scenario over the last millennia, because it is unlikely that an event significantly stronger than that had occurred during the entire Holocene.

### Statistics

Model statistical significance is tested using a Student *t*-test^43^ with the null hypothesis that the difference in means between the runs with and without the event is not significantly different from zero. The significance of the correlation coefficients was estimated by the non-parametric random-phase method^44^. Model uncertainties (1σ) were calculated from the ensemble members and represent the internal variability of the model.

## Additional Information

**How to cite this article:** Sukhodolov, T. *et al*. Atmospheric impacts of the strongest known solar particle storm of 775 AD. *Sci. Rep.* **7**, 45257; doi: 10.1038/srep45257 (2017).

**Publisher's note:** Springer Nature remains neutral with regard to jurisdictional claims in published maps and institutional affiliations.

## Supplementary Material

Supplementary Information
