# Supplementary material for: Atmospheric impacts of the strongest known solar particle storm of 775 AD
Source: Sci Rep. 2017 Mar 28;7:45257. doi: 10.1038/srep45257 (PMC5368659; doi:10.1038/srep45257)
Supplement: Supplementary Information [file srep45257-s1.doc]

**Supplementary material for the manuscrict “Atmospheric impacts of the strongest known solar particle storm of 775 AD”**

**Timofei Sukhodolov*^1,2^, Ilya Usoskin^3,4^, Eugene Rozanov^1,2^, Eleanna Asvestari^3^, William Ball^1,2^, Mark A. J. Curran^5,6^, Hubertus Fischer^7^, Gennady Kovaltsov^8^, Fusa Miyake^9^, Thomas Peter^2^, Christopher Plummer^6,10^, Werner Schmutz^1^, Mirko Severi^11^, Rita Traversi^11^.**

^1^Physikalisch-Meteorologisches Observatorium Davos World Radiation Centre, Davos, Switzerland

^2^Institute for Atmospheric and Climate Science, Swiss Federal Institute of Technology Zurich, Zurich, Switzerland

^3^Space Climate Research group, University of Oulu, Finland

^4^Sodankylä Geophysical Observatory, University of Oulu, Finland

^5^Department of the Environment, Australian Antarctic Division, Kingston, Australia

^6^Antarctic Climate and Ecosystem Cooperative Research Centre, University of Tasmania, Hobart, Australia

^7^Climate and Environmental Physics, Physics Institute and Oeschger Centre for Climate Change Research, University of Bern, Bern, Switzerland^8^Ioffe Physical-Technical Institute RAS, St. Petersburg, Russia

^9^Institute for Space-Earth Environmental Research, Nagoya University, Nagoya, Japan

^10^Institute for Marine and Antarctic Studies, University of Tasmania, Hobart, Australia

^11^Dept. of Chemistry “Ugo Schiff”, University of Florence, Florence, Italy

**
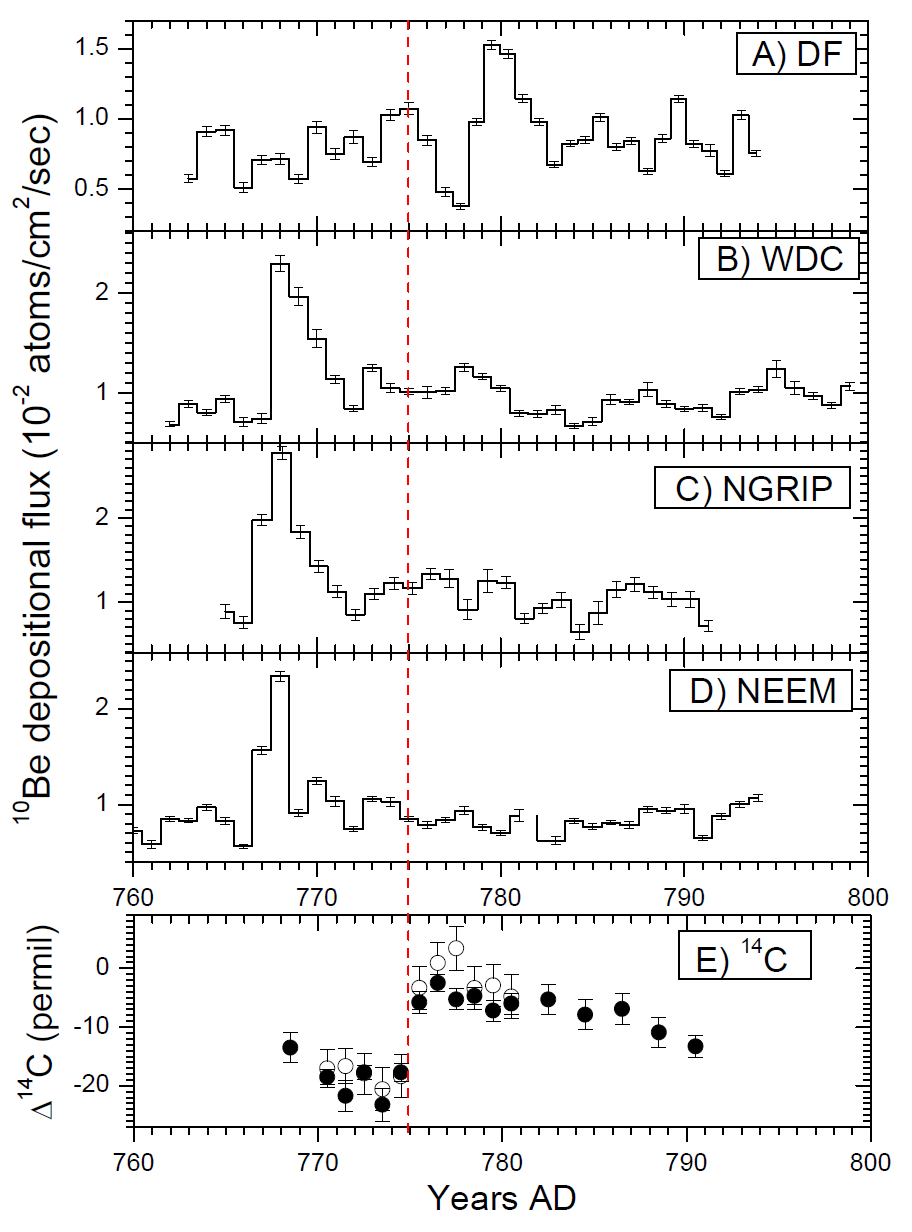
**

**Figure S1:** Annual data of cosmogenic isotopes for the period around 775 AD. Panels A through D correspond to the depositional fluxes of ^10^Be measured in four different sites: Dome Fuji (Antarctica), WAIS/WDC (Antarctica), NGRIP (Greenland) and NEEM (Greenland), respectively (Table S1) with the original uncorrected dating^10-11^. Panel E depicts the radiocarbon content Δ^14^C in tree rings for the Japanese cedar^5^ (solid dots) and German oak^6^ (open circles). The vertical red dashed line marks the year 775 AD when the event took place, according to absolutely dendrochronologically dated ^14^C data^5^.

**Figure S2:** Annual depositional fluxes of ^10^Be at four sites analyzed here for the period around 775 AD. Panels A through D correspond to the sites of Dome Fuji (Antarctica), WDC (Antarctica), NGRIP (Greenland) and NEEM (Greenland), respectively. Red lines with error bars depict the measured data, while other lines depict the modeled ^10^Be flux for the four scenarios (See Table S2). Years are given relative to the peak year. See Tables S1-S2 in SM for the sites and scenarios details.

**Figure S3:** Modelled changes of the HNO_3_ wet and dry deposition (%) due to the event on 01-Sep-774 averaged over the first month after the event. Colored areas are significant at a 95% confidence level. (Maps are plotted using IDL version 8.2, http://www.harrisgeospatial.com/ProductsandSolutions/GeospatialProducts/IDL.aspx).

**Figure S4:** Monthly mean total ozone differences between numerical experiments with and without the event. Panel A: zonal mean changes in Dobson units (the thickness of the layer which would be formed by the total gas in a column under standard temperature and pressure), colored areas are significant at the 95% confidence level. Panel B: global mean changes (%), shaded area represents 1σ uncertainty.

**Figure S5:** Simulated annual zonal mean concentration of ^10^Be in atoms/cm^3^ produced by GCRs for the year 774 AD (ϕ ~ 600 MV). The orange line indicates the average position of the tropopause. Tropospheric asymmetry is related to the stronger stratosphere-troposphere coupling in the northern hemisphere and the fact that the deposition takes place only in the lowest model layer.

**Table S1.** Parameters of the sites with ice core data of ^10^Be and nitrate used in this study: Name of the core and reference to the data source; geographical coordinates and elevation; the year *Y_o_* corresponding to the peak.

| Ice core | Data | Coordinates | Elevation | Method | *Y_o_* |
| --- | --- | --- | --- | --- | --- |
| West Antarctic Ice Sheet (WAIS) Divide Ice Core (WDC)^9,10^ | ^10^Be | 79.48^o^S 112.11^o^W | 1759 m | AMS | 768 AD |
| Dome Fuji (DF), Antarctica^11^ | ^10^Be | 77.3^o^S 39.7^o^E | 3810 m | AMS | 780 AD |
| North Greenland Ice Core Project (NGRIP)^9,10^ | ^10^Be | 75.1^o^N 42.32^o^W | 2917 m | AMS | 768 AD |
| North Greenland Eemian Ice Drilling (NEEM) ^9,10^ | ^10^Be and NO_3_^-^ | 77.45^o^N 51.06^o^W | 2450 m | AMS, CFA | 768 AD |
| Law Dome (LD), Antarctica^39^ | NO_3_^-^ | 66.73°S 112.83°E | 1370 m | ICM | - |
| Talos Dome (TD), Antarctica^41^ | NO_3_^-^ | 73°S 158°E | 2318 m | FIC | - |
| European Project for Ice Coring in Antarctica (EPICA) Dome C (EDC), Antarctica^41^ | NO_3_^-^ | 75.1°S 123.3°E | 3233 m | FIC | - |
| Greenland Ice Sheet Project (GISP2)^45^ | NO_3_^-^ | 72.36°N 38.30°W | 3200 m | IC | - |

**Table S2.** Comparison of the model results in Scenarios 1-4 (Sc1-Sc4) with the measured fluxes of ^10^Be for four sites as described in the text. Shown are: the scaling factor *f,* the integral deposition (in 10^7^ at/cm^2^) of the ^10^Be due to the event, as measured *I* and modelled *I_mod_*, the ratio of the modelled to measured integrals over the peak *R=I_mod_/I*; the Pearson's correlation *C* and its significance *p* (in parentheses) for the years -1 through 3. Colors indicate highly significant (*p*<0.01, green), significant (*p*<0.05, yellow) and insignificant (*p*>0.05, red) correlations.

| Site and parameter | Sc1 (June 774) | Sc2 (Sept. 774) | Sc3 (Dec. 774) | Sc4 (March 775) |
| --- | --- | --- | --- | --- |
| Site and scaling *f* | Dome Fuji (*f* = 1.42±0.07, I=6.31 ) | | | |
| *I_mod_* | 6.63 | 6.31 | 6.03 | 6.66 |
| *R* | 1.05±0.03 | 1.00±0.03 | 0.96±0.03 | 1.06±0.03 |
| *C_p_* | 0.88(0.04) | 0.99(0.001) | 0.92(0.026) | 0.96(0.011) |
| Site | WAIS/WDC (*f* = 1.88±0.11, I=8.15) | | | |
| *I_mod_* | 7.44 | 7.30 | 7.12 | 7.39 |
| *R* | 0.91±0.04 | 0.90±0.04 | 0.87±0.04 | 0.91±0.04 |
| *C_p_* | 0.93(0.022) | 0.98(0.003) | 0.96(0.011) | 0.94(0.017) |
| Site | NGRIP (*f* = 1.00±0.06, I=11.6) | | | |
| *I_mod_* | 9.50 | 10.5 | 10.8 | 9.56 |
| *R* | 0.82±0.04 | 0.91±0.04 | 0.93±0.04 | 0.83±0.04 |
| *C_p_* | 0.90(0.037) | 0.88(0.04) | 0.98(0.003) | 0.78(0.12) |
| Site | NEEM (*f* = 0.79±0.04, I=9.21) | | | |
| *I_mod_* | 7.64 | 7.89 | 8.20 | 7.23 |
| *R* | 0.83±0.04 | 0.86±0.04 | 0.89±0.04 | 0.79±0.04 |
| *C_p_* | 0.81(0.1) | 0.89(0.04) | 0.91(0.03) | 0.67(0.22) |
